# Comparison of leucocyte profiles between healthy children and those with asymptomatic and symptomatic *Plasmodium falciparum* infections

**DOI:** 10.1186/s12936-020-03435-x

**Published:** 2020-10-09

**Authors:** Diana Ahu Prah, Linda Eva Amoah, Matthew P. Gibbins, Yaw Bediako, Aubrey J. Cunnington, Gordon A. Awandare, Julius Clemence R. Hafalla

**Affiliations:** 1 West African Centre for Cell Biology of Infectious Pathogens, Department of Biochemistry, Cell and Molecular Biology, University of Ghana, Legon, Ghana; 2 Department of Infection Biology, Faculty of Infectious and Tropical Medicine, London School of Hygiene and Tropical Medicine, London, UK; 3 Immunology Department, Noguchi Memorial Institute for Medical Research, University of Ghana, Legon, Ghana; 4 Section of Paediatrics, Imperial College, London, UK; 5 Present Address: Wellcome Centre for Integrative Parasitology, Institute of Infection, Immunity and Inflammation,, University of Glasgow, Glasgow, UK

**Keywords:** Malaria, *Plasmodium falciparum*, Symptomatic, Asymptomatic, Leucocytes, Neutrophils, Immunity, Pathogenesis

## Abstract

**Background:**

The immune mechanisms that determine whether a *Plasmodium falciparum* infection would be symptomatic or asymptomatic are not fully understood. Several studies have been carried out to characterize the associations between disease outcomes and leucocyte numbers. However, the majority of these studies have been conducted in adults with acute uncomplicated malaria, despite children being the most vulnerable group.

**Methods:**

Peripheral blood leucocyte subpopulations were characterized in children with acute uncomplicated (symptomatic; n = 25) or asymptomatic (n = 67) *P. falciparum* malaria, as well as malaria-free (uninfected) children (n = 16) from Obom, a sub-district of Accra, Ghana. Leucocyte subpopulations were enumerated by flow cytometry and correlated with two measures of parasite load: (a) plasma levels of *P. falciparum* histidine-rich protein 2 (*Pf*HRP2) as a proxy for parasite biomass and (b) peripheral blood parasite densities determined by microscopy.

**Results:**

In children with symptomatic *P. falciparum* infections, the proportions and absolute cell counts of total (CD3 +) T cells, CD4 + T cells, CD8 + T cells, CD19 + B cells and CD11c + dendritic cells (DCs) were significantly lower as compared to asymptomatic *P. falciparum-*infected and uninfected children. Notably, CD15 + neutrophil proportions and cell counts were significantly increased in symptomatic children. There was no significant difference in the proportions and absolute counts of CD14 + monocytes amongst the three study groups. As expected, measures of parasite load were significantly higher in symptomatic cases. Remarkably, *Pf*HRP2 levels and parasite densities negatively correlated with both the proportions and absolute numbers of peripheral leucocyte subsets: CD3 + T, CD4 + T, CD8 + T, CD19 + B, CD56 + NK, γδ + T and CD11c + cells. In contrast, both *Pf*HRP2 levels and parasite densities positively correlated with the proportions and absolute numbers of CD15 + cells.

**Conclusions:**

Symptomatic *P. falciparum* infection is correlated with an increase in the levels of peripheral blood neutrophils, indicating a role for this cell type in disease pathogenesis. Parasite load is a key determinant of peripheral cell numbers during malaria infections.

## Background

Malaria, caused by *Plasmodium falciparum*, continues to cause significant morbidity and mortality particularly in young children from sub-Saharan Africa [1]. Despite advances in control and elimination efforts, malaria parasites and the mosquitoes that transmit them are increasingly developing resistance to currently available drugs and insecticides, respectively [2–4]. Novel control measures are urgently warranted in the global fight against malaria. The development of alternative clinical interventions is hindered by the limited understanding of malaria pathogenesis, particularly the complex interactions between the parasite and the host's immune system.

After mosquito inoculation, malaria sporozoites infect the liver, where the parasites replicate silently. The subsequent blood-stage infection leads to acute uncomplicated febrile illness, which can progress to severe life-threatening disease, as the parasite load increases [5]. However, individuals who survive and are repeatedly re-infected acquire clinical immunity, which limits parasite load and thereby reduces the likelihood of developing clinical symptoms [6]. Since naturally acquired immunity does not completely clear the parasites, individuals remain susceptible to asymptomatic infections [7]. Typically, the highest parasite loads are seen in individuals with severe malaria, lower in symptomatic uncomplicated malaria, and lowest (often-times only positive by molecular methods) in asymptomatic infections. Yet, there is overlap in the parasite loads amongst these groups, such that some individuals with relatively high parasite load can be asymptomatic and others with relatively low parasite load can develop symptoms or even severe complications [8]. The mechanisms of disease progression remain poorly understood and it is likely that there is either constitutive or acquired variation in the ability of individuals to tolerate a given parasite load without symptoms.

Blood-stage infection causes all of the pathological consequences of malaria, suggesting that alteration of any blood parameters could directly influence the risk of disease manifestations [9]. Immune cells like T (CD3 +) cells and natural killer (NK) cells are known to play crucial roles in the immune response generated against pathogens. CD4 + T cells and NK cells produce pro-inflammatory cytokines, such as interferon-γ (IFN-γ), that promote parasite killing and clearance of infected erythrocytes by activating monocytes and macrophages, and enhancing B cell function [10–12]. The function of CD8 + T cells during *P. falciparum* blood-stage infection is poorly understood, although recent data suggest their role in severe diseases, such as cerebral malaria [13]. γδ T cells are known to expand following malaria infection and correlate with protection from infection, but this response is attenuated with repeated exposure [14]. Activated B-cells produce antibodies that play vital roles in controlling the blood-stage parasites. Effector functions of antibodies include inhibition of merozoites invasion of erythrocytes [15], opsonization of infected erythrocytes for rapid clearance by macrophages [16], and antibody-dependent cytotoxicity and cellular killing (ADCC) of parasites by natural killer (NK) cells [17]; the underlying mechanism for the latter remains unclear. It is now widely accepted that endemic individuals have antibodies against a broad range of parasites antigens [18], yet these individuals remain susceptible to infection. In addition to contributing to the protection, activation of immune cells, when dysregulated, also promote disease pathogenesis. While neutrophils can mediate parasite killing when activated [19], they also release molecules such as proteases or neutrophil extracellular traps (NETs), which can also be very destructive to host tissues [20, 21]. Pro-inflammatory mediators, such as IFN-γ and tumor necrosis factor (TNF), that support parasite killing and clearance can cause uncontrolled inflammation and lead to the sequestration of infected erythrocytes. Thus, the regulation of inflammatory responses, orchestrated by CD4 + T cells and monocytes/macrophages, is key to the successful resolution of malaria blood-stage infection [22, 23].

Changes in peripheral blood leucocyte counts during *P. falciparum* infection have been described. The reduction in the percentages and absolute cell counts of the major lymphocyte populations (total T cells, CD4 + T helper cells, CD8 + cytotoxic T cells, and B cells) and NK cells seems to be a feature of acute malaria infection [24–27]. However, there are also conflicting reports, where no differences in the percentages of T cell subsets were identified during acute mild malaria relative to uninfected control groups [28, 29]. It has also been suggested that factors such as age, sex, geographical location (linked to transmission intensity) and host genetic factors may alter the distribution of T cell populations during acute infection [25]. For example, γδ T cells were found to rapidly expand following acute *P. falciparum* infection in adults [24], with no evidence of an increase in the numbers of this cell type in children [28].

Despite the insights gained from previous studies, it is striking that the majority of these reports have only focused on contrasting symptomatic and uninfected cases. Very few studies have considered asymptomatic individuals. Since asymptomatic parasite carriers are clinically immune, they might have immune responses that differ from symptomatic individuals. Studies that compare symptomatic and asymptomatic children of similar age could provide insight into the nature of immune responses that mediate both resistance and tolerance to parasites. This present work addresses this question by profiling leucocyte subpopulations in Ghanaian children with symptomatic and asymptomatic *P. falciparum* infection and assessing the relationship between cell numbers and measures of parasite load.

## Methods

### Ethics approval and consent to participate

The collection of human samples used in this study was approved by the ethical committees of the Noguchi Memorial Institute for Medical Research, University of Ghana (No. 024/14-15) and the London School of Hygiene and Tropical Medicine (ID Nos. 14322, 15684 and 17257). Written informed consent was obtained from parents or guardians and assent appropriately received from the children before they were enrolled in this study.

### Study site, participants and sampling

This was a cross-sectional study conducted in Obom, a sub-district of Accra, Ghana. Obom is hyperendemic for malaria [30], and samples were collected throughout the whole year. We enrolled children (6–12 years) who were either symptomatic for malaria (n = 25), asymptomatic (n = 67) or uninfected (n = 16). The groups were matched as closely as possible for age and gender. Symptomatic participants included children who attended the Obom Community Health Centre with malaria-related symptoms and were confirmed to have parasites by microscopy. For the asymptomatic and the uninfected groups, individuals were identified by screening apparently healthy children in basic schools. Children found to have parasites by microscopy and/or PCR were included in the asymptomatic group, whereas those without parasites were designated as uninfected controls. Children were not included if they had used any medication 2 weeks before sampling or had any condition that could interfere with the experimental outcome, such as non-productive cough, or sickle cell disease. Asymptomatic children were followed for one week after the initial screening with the aim to exclude those individuals who developed symptoms (pre-symptomatic); none developed symptoms within this period. Peripheral blood samples (5 ml) were collected into EDTA and PAXgene tubes before any anti-malarial treatment and were transported at 4 °C to the laboratory for investigation. Samples were collected before symptomatic children received treatment at the health centre. A complete blood count (BC-5200 Haematology Analyser), sickle cell screen (sodium metabisulfite test) and glucose-6-phosphate dehydrogenase (G6PD) deficiency test (fluorescent spot test (FST; Procedure 203, Trinity Biotech, Ireland) were performed for each sample.

### Parasite identification

Thick/thin blood smears and filter paper dried blood spots (DBS) were prepared for microscopy and PCR, respectively. Smears were stained with Giemsa and examined blindly for the presence of parasites by two independent expert microscopists. Parasite density in the blood was calculated by assuming a white blood count (WBC) of 8000/µl and at least 500 fields were examined by microscopy before a smear was regarded as negative. For the PCR analysis, the DBS were lysed overnight with 1X HEPES buffered saline (HBS) and 0.5% Saponin, and DNA samples were extracted and used for parasite detection [31]. The *Pf*HRP2 ELISA assay (Malaria Ag ELISA; Cellabs Pty. Ltd., Brookvale, New South Wales, Australia) was performed as a proxy for total parasite biomass. A standard curve was generated from the positive control included in the kit and this was used to calculate the *Pf*HRP2 levels in the study samples.

### Whole blood flow cytometry

#### Antibodies used for flow cytometry

Anti-human antibodies used to identify the various leucocyte subpopulations were: CD3-BUV 737 (clone SK7), CD19-PE-Cy7 (clone HIB19), CD11c-BUV 395 (clone B-ly6) (BD Biosciences); CD4-AF700 (clone RPA-T4) (eBioscience); CD8-FITC (clone HIT8a), CD14-APC (clone M5E2), CD56-PE (clone HCD56), γδ-PE-CF594 (clone B1), and CD15-PerCP-Cy5.5 (clone W6D3) (BioLegend). eFluor™ 780 fixable viability dye (Invitrogen) was included to exclude dead cells from the data. All antibodies were titrated to determine the optimal concentrations.

#### Cell surface staining and flow cytometric analysis

Freshly drawn whole blood samples (100 ul) were incubated with a premixed antibody cocktail in a final staining volume of 120 µl for 20 min at room temperature in the dark. After incubation, samples were washed with 2 ml staining buffer (0.2% w/v BSA, 1X PBS and 0.1% NaN3). Red blood cells were lysed with 2 ml of 1X red blood cell lysis solution (BD Biosciences, Oxford, UK) for 15 min at room temperature in the dark. Following 2 washes, samples were fixed in 4% paraformaldehyde for 15 min in the dark, washed and resuspended in 0.5 ml of staining buffer and immediately examined using BD Fortessa by recording 200,000 or more events. Instrument calibration was checked daily by use of BD FACSDivaTM CS&T beads. Results were compensated and analysed using Flowjo software (version 10.07 for Windows, TreeStar, Ashland, USA). Single colour stained compensation was prepared to set gates. Except for the fixable viability dye where whole blood samples were used, single stained compensation controls were prepared with BD CompBead Plus (CD4 + and CD8 +) and BD CompBead (for the remaining markers). The gating strategies are outlined in Additional file 1: Fig. S1. The absolute cell counts of leucocyte subpopulations were obtained by multiplying the absolute WBC count determined with an automated haematology analyser (Table 1) by the total percentage of each cell population determined by flow cytometry.

**Table 1:** Demographic characteristics and haematological indices of the study population

Variables	Symptomatic N = 25	Asymptomatic N = 67	Uninfected control N = 16	*P* value
Age (years)	9 (7–12)	9(8–11)	10 (8.25–11)	0.7621^a^
Gender (%), Female/Male	48/52	43/57	62/38	0.3830^b^
Parasite density (/µl),	13,893 (4762–36,300)	400(160–630)^20^	NA	< 0.0001^c^
*Pf*HRP2 (ng/ml)	291.6 (7.87–1100)^22^	6.44 (2.42—12.73)^29^	NA	< 0.0001^c^
Haematological indices				
Haemoglobin (g/dl)	11.00 (8.50–12.00)	10.70 (10.00–12.00)	11.70 (10.33–12.88)	0.0828^a^
WBC (× 10^9^/l)	6.60 (4.93–7.99)	6.20 (4.82–7.38)	6.98(6.20–8.73)	0.2511^a^
Neutrophils (× 10^9^/l)	4.3 (3.04–5.70)	1.84(1.40–2.30)	1.84(0.87–2.55)	< 0.0001^a^
Neutrophils (%)	69.90 (61.45–78.25)	29.50 (23.50–38.70)	25.35 (21.63–32.18)	< 0.0001^a^
Lymphocytes (× 10^9^/l)	1.00 (0.70–1.86)	3.37 (2.59–4.30)	4.19 (3.13–4.58)	< 0.0001^a^
Lymphocytes (%)	17.90 (11.20–28.75)	55.90 (47.60–67.68)	59.00 (49.00–67.68)	< 0.0001^a^
Monocytes (× 10^9^/l)	0.40 (0.24–0.65)	0.52(0.34–0.72)	0.52 (0.44–0.62)	0.3366^a^
Monocytes (%)	7.30 (3.95–10.47)	8.10 (6.40–10.70)	8.00 (6.83–9.13)	0.4395^a^
Platelets (× 10^9^/l)	109 (59.0–144.0)	264 (205.0–334.0)	241.0 (204.8–334.8)	< 0.0001^a^

*PfHRP2* *P. falciparum* histidine-rich protein 2; *WBC* white blood cell count. Data are median [interquartile range (IQR)], and superscripts indicate the number of subjects with data for each variable if less than the total. ^a^Kruskal–Wallis test, ^b^Chi-square test, ^c^Mann–Whitney U test

### Statistical analysis

All statistical analyses were conducted in GraphPad Prism 8.1.2. The data relating to demographics and haematological parameters of the study subjects were compared amongst the study groups using the two-tailed non-parametric Kruskal–Wallis test with Dunn’s post hoc test for multiple comparisons of the leucocyte subpopulation amongst the study groups, and Chi-square test for categorical variables. Box and whiskers plot using the Tukey method was used to represent figures. Briefly, the difference between the 25th and 75th percentile (IQR: interquartile range) was calculated for each group. If the sum of the 75^th^ percentile and 1.5*IQR is greater than or equal to the largest value in the data set, the upper whisker is drawn to this largest value. Otherwise, the upper whisker is set to the largest value less than the sum, and values larger than this are plotted as individual points. If the difference between the 25th percentile and 1.5*IQR is less than or equal to the smallest value in the data set, the lower whisker is then drawn to this smallest value. If not, the lower whisker is set to the lowest value greater than the difference, and values less than this are plotted as individual points. The Mann Whitney test was used to compare measures of parasite load between symptomatic and asymptomatic groups. The non-parametric Spearman’s rank correlation was used to assess the association between leucocyte subpopulations and measures of parasite load. Values of *p* < 0.05 were considered statistically significant. The GLM function in R was used to construct multivariable models to investigate the association between measures of parasite load and leucocyte counts or proportions, age and gender. For the purpose of these models, *Pf*HRP2, parasite density and absolute cell counts were log-normalized.

## Results

### Characteristics of the study population

One hundred and eight children were enrolled from the Obom community: 25 with symptomatic malaria, 67 with asymptomatic infections and 16 uninfected controls. The demographic, haematological and microbiological characteristics of the study participants are shown in Table 1. The median age was 9.5 years (range 6–12) and 47% were females. Subjects were well matched for age and sex between groups. As expected, plasma *Pf*HRP2 levels and parasite densities were significantly higher in symptomatic individuals than asymptomatic individuals (*p* < 0.0001) (Table 1). In the asymptomatic participants, all subjects had a positive *Pf* PCR result, 43% had detectable *Pf*HRP2 by ELISA, and 30% had detectable parasites by microscopy. All symptomatic participants were positive by microscopy.

Notably, the haematological indices did not differ significantly between the asymptomatic and the uninfected control groups. Although haemoglobin concentrations (*p* = 0.0828), total white blood cell counts (*p* = 0.2511) and monocytes (*p* = 0.3372) were relatively similar amongst the groups, there were marked differences in some of the constituent leucocyte subpopulations and platelet count. Neutrophil counts per microlitre of blood (*p* < 0.0001) and neutrophil proportions (*p* < 0.0001) were significantly higher in the symptomatic group compared to asymptomatic and uninfected groups (Table 1). Moreover, platelet counts (*p* < 0.0001) and total lymphocytes proportions (*p* < 0.0001) for the symptomatic group were significantly lower than those measured in both the asymptomatic and uninfected controls.

### Children with symptomatic P. falciparum infection display significant changes in peripheral blood lymphocyte subsets compared to asymptomatic and healthy children

**Fig. 1:**
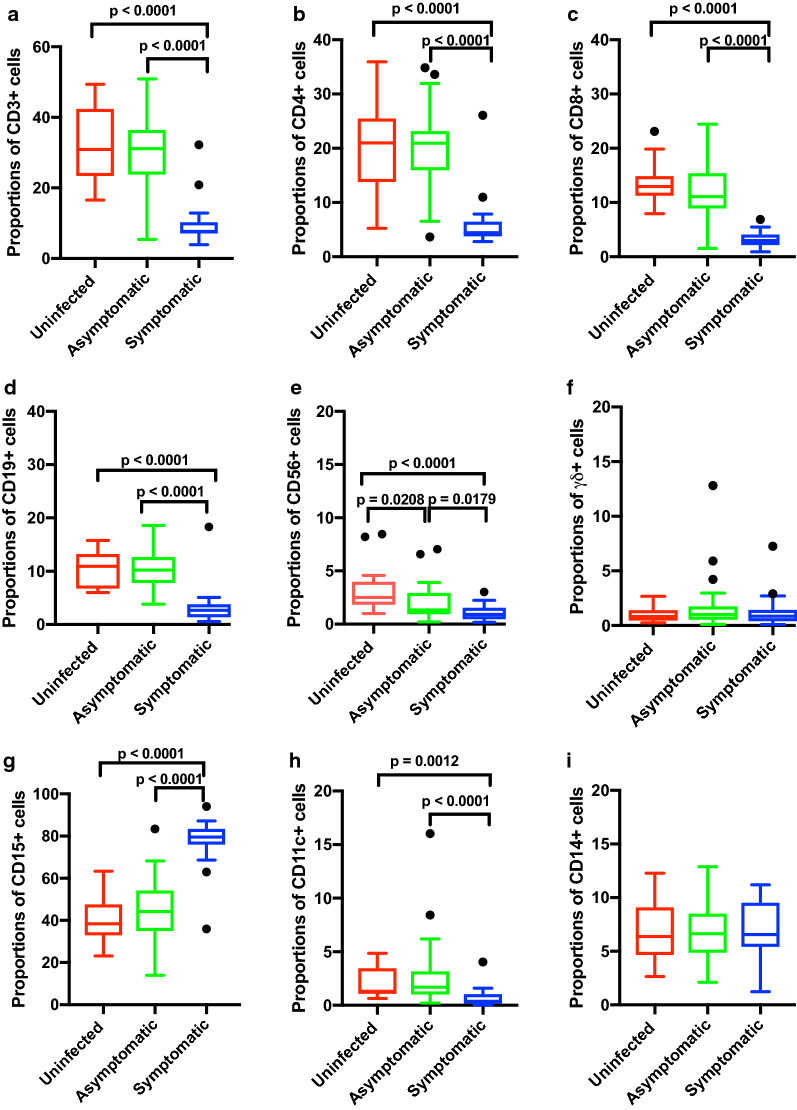
Changes in the proportions of leucocytes in the peripheral blood of Ghanaian children who are uninfected (n = 15), or with asymptomatic (n = 52) or symptomatic (n = 22) *P. falciparum* infections.** a** Total CD3 + T cells, **b** CD4 + T cells, **c** CD8 + T cells, **d** CD19 + B-cells, **e** CD56 + NK cells, **f** γδ + T cells, **g** CD15 + neutrophils, **h** CD14 + monocytes and **i** CD11c + DCs. Data are represented as box and whiskers plots using theTukey method; Kruskal–Wallis test followed by Dunn’s test

Multicolour flow cytometry was utilized to identify and enumerate the proportions of peripheral blood total (CD3 +), CD4 + and CD8 + T cells, CD19 + B cells, CD56 + NK and γδ T cells in the three groups of participants. Flow cytometry data were available for 22 symptomatic, 52 asymptomatic and 15 uninfected participants. Analyses were not performed for other samples because they were kept overnight at 4 °C before being transported to the laboratory. The gating strategy used to analyse the flow cytometry data is shown as a supplementary material (Additional file 1: Fig. S1). Compared to those with asymptomatic infections and the uninfected group, children with symptomatic malaria demonstrated a significant reduction in the proportions (Fig. 1a–f) and absolute numbers (Additional file 2: Fig. S2a-f) of peripheral blood total T cells, CD4 + and CD8 + T cells, and CD19 + B cells (p < 0.0001 for all comparisons). The proportions and absolute counts of CD56 + NK cells were significantly higher in the uninfected compared to the asymptomatic (p = 0.0054 for absolute counts and p = 0.0208 proportions) or symptomatic (p < 0.0001 for both comparisons) groups. Although there was no significant difference (p = 0.0690) in the absolute numbers of CD56 + NK cells between asymptomatic and symptomatic children, the proportions were significantly lower in the symptomatic group (p = 0.0179). γδ T cell absolute numbers only differed between the uninfected and the symptomatic groups (p = 0.0480).

### Circulating neutrophils are increased during acute malaria in children

A comparison of myeloid-derived proportions (Fig. 1g–i) and cell numbers (Additional file 2: Fig. S2g–i) in the peripheral blood was also made amongst the three groups. Consistent with haematological data, we observed a significant increase in the proportions and absolute numbers of CD15 + neutrophils in the symptomatic children compared to the asymptomatic (*p* < 0.0001 for both comparisons) and uninfected (p < 0.0001 proportions and *p* = 0.0035 for absolute counts) groups. The proportions and absolute numbers (p > 0.05 for both comparisons) of CD15 + cells were not significantly different between the uninfected and asymptomatic groups. The changes in the proportions and absolute numbers of CD11c + DCs mirrored the data of lymphocytes, where a significant decrease was found in the symptomatic groups as compared to the uninfected and asymptomatic groups (*p* < 0.0001 for all comparisons); no significant difference was found between the asymptomatic and the uninfected control group (*p* > 0.05). There were no significant differences in CD14 + monocytes amongst the groups (*p* > 0.05 for comparisons of both proportions and absolute numbers amongst groups).

### Relationship between parasite load and leucocyte subpopulations

High levels of parasites are typically associated with an increased risk of developing severe disease (8). Since the proportions and absolute numbers of peripheral leucocyte populations seem to influence disease outcome, it was determined whether there was any relationship between parasite load and the leucocyte subpopulations quantified in this study. For this purpose, only individuals (asymptomatic or symptomatic) with detectable parasites by microscopy (parasite density counts) or detectable plasma *Pf*HRP2 concentrations (indicating total parasite biomass) were included in this analysis. For the asymptomatic group, only 20 had positive parasite density data and 29 participants had plasma *Pf*HRP2 levels above the detection limit. For the symptomatic group, 22 participants had data for both parasite density and plasma *Pf*HRP2. Considering the combined available data for measures of parasite load between symptomatic and asymptomatic children, a strong positive correlation was observed between *Pf*HRP2 levels and parasite densities (*r* = 0.7074, *p* < 0.0001).

**Fig. 2:**
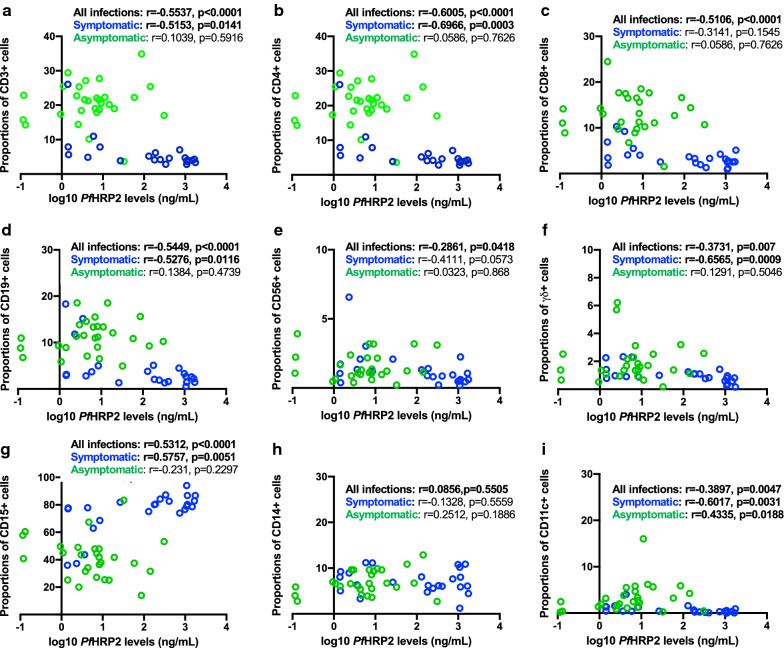
Relationships between parasite biomass (*Pf*HRP2 plasma levels) and proportions of different cell subsets in the peripheral blood of *Pf*-infected children. Data (51 total) includes both symptomatic (n = 22, blue circles) and asymptomatic (n = 29, green circles) children. **a** Total CD3 + T cells, **b** CD4 + T cells, **c** CD8 + T cells, **d** CD19 + B-cells, **e** CD56 + NK cells, **f** γδ + T cells, **g** CD15 + neutrophils, **h** CD14 + monocytes and **i** CD11c + DCs. Data analysed for all values (as well as for symptomatics and asymptomatics) using a Spearman correlation coefficient test; r and p values are shown

Correlations between proportions of the different leucocytes and parasite load for all subjects combined are shown in Fig. 2 (against *Pf*HRP2 levels) and 3 (against parasite density). Correlations between absolute counts of the different leucocyte populations and parasite load for all subjects combined are shown in Additional file 3: Fig. S3 (against *Pf*HRP2 levels) and S4 (against parasite density). For ease of visualization, data points are coloured green and blue on the graphs, denoting asymptomatic and symptomatic children, respectively. Notably, based on these analyses, the proportions and absolute numbers of total T cells, CD4 + T cells, CD8 + T cells, CD19 + B cells, CD11c + DCs, CD56 + NK cells and γδ + T cells inversely correlated with both *Pf*HRP2 levels and parasite density (see Figs. for r and p values). Conversely, there was a strong positive correlation between the frequency of CD15 + neutrophils and the two measures of parasite load. No correlation was observed with CD14 + monocytes.

**Fig. 3:**
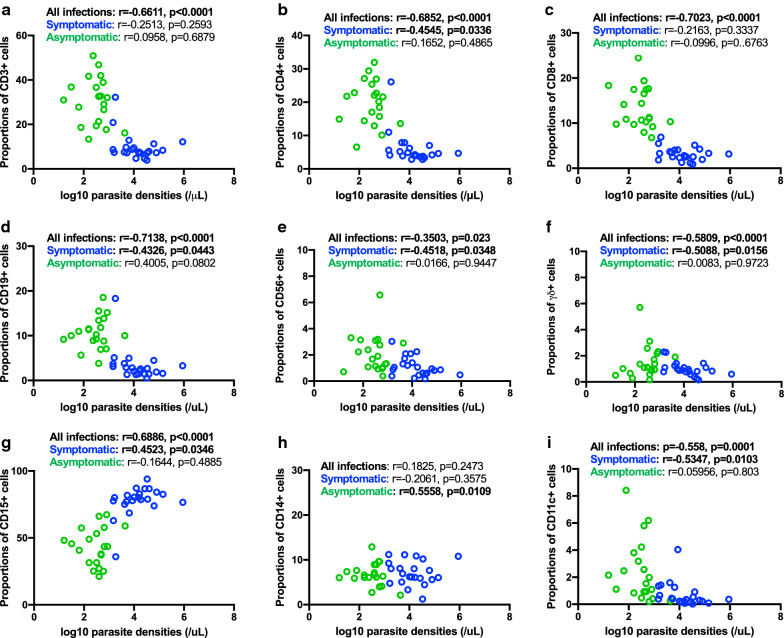
Relationships between parasite densities and proportions of different cell subsets in the peripheral blood of *Pf*-infected children. Data (51 total) includes both symptomatic (n = 22, blue circles) and asymptomatic (n = 20, green circles) children. **a** Total CD3 + T cells, **b** CD4 + T cells, **c** CD8 + T cells, **d** CD19 + B-cells, **e** CD56 + NK cells, **f** γδ + T cells, **g** CD15 + neutrophils, **h** CD14 + monocytes and **i** CD11c + DCs. Data analysed for all values (as well as for symptomatics and asymptomatics) using a Spearman correlation coefficient test; *r* and *p* values are shown

Further analysis was performed to determine whether these relationships between parasite load and the leucocyte subpopulations were affected by disease severity, by comparing the asymptomatic and symptomatic groups separately. The analyses are shown after the calculation of overall correlations (Figs. 2, 3, Additional file 3: Fig. S3 and Additional file 4: Fig. S4). For the symptomatic group, the proportions of total T cells (p = 0.0141), CD4 T cells (p = 0.0003), CD19 + B cells (*p* = 0.0116), CD11c DC cells (*p* = 0.0031) and γδ + T cells (p = 0.0009) negatively correlated with *Pf*HRP2 levels, whereas CD15 + neutrophils showed a positive correlation (*p *= 0.0051). The absolute numbers of CD11c + DCs negatively correlated (*p* = 0.0108) and CD15 + neutrophils positively correlated (*p* = 0.0129) with *Pf*HRP2 levels. In the case of parasite density, there was a significant negative correlation between the absolute numbers of CD56 + NK cells (p = 0.0453) and CD11c + DCs positively correlated (*p *= 0.0089). The proportions of CD4 + T cells (*p* = 0.0336), CD19 + B cells (*p* = 0.0443), CD56 + NK cells (p = 0.0348), γδ + T cells (*p* = 0.0156) and CD11 + DCs (*p* = 0.0103) negatively correlated with parasite densities, whereas a positive correlation for CD15 + neutrophils was obtained (*p* = 0.0346). Very few significant correlations with data from asymptomatic participants were obtained. Against *Pf*HRP2 levels, only the absolute numbers (*p* = 0.0388) and the proportions (*p* = 0.0188) of CD11c + DCs showed a positive correlation. Against parasite densities, only the absolute numbers (*p* = 0.0296) and the proportions (*p* = 0.0109) of CD14 + monocytes showed a positive correlation.

Finally, an exploratory multivariable analysis was performed to investigate whether age and gender influenced each of the associations we observed between measures of parasite load (PfHRP2 or parasite density) and cell proportions or absolute counts, in the symptomatic or asymptomatic subjects. None of the resulting models showed significant (*P* < 0.05) effects of age and sex on the associations between leucocytes and parasite load.

## Discussion

Numerous studies have indicated that *Plasmodium* infection impacts the frequencies of peripheral blood leucocyte subsets. Yet, the majority of these studies have focused exclusively on symptomatic paired healthy cases. The present study was undertaken to address the limited information on the differences between the peripheral blood leucocyte profiles of children with symptomatic and asymptomatic *P. falciparum* infections. Here, both the proportions (Figs. 1, 2, 3) and absolute counts (Additional file 2: Fig. S2, Additional file 3: Fig. S3, Additional file 4: Fig. S4) of different leucocyte populations were reported. Despite the outcomes for both were comparable in the present study, it is important to emphasize that absolute cell counts of leucocyte subpopulations are independent of each other, whereas proportions are not. It is conceivable that if the proportion of one cell type increases, one or more of the others decreases.

The present study showed that symptomatic infections were associated with decreased proportions and absolute counts of total CD3 + T cells, CD4 + and CD8 + T cells, CD19 + B cells, and CD11c + cells compared to asymptomatic infections, corroborating previously reported data [25, 27]. The numbers of peripheral blood CD14 + and γδ + cell counts were not significantly different amongst the groups. Notably, the levels of CD15 + neutrophils were significantly higher in children with acute symptomatic malaria, and the numbers positively correlated with measures of parasite load, suggesting a causal link. The results also indicate that the cellular compositions of peripheral blood in children with asymptomatic malaria infections are very similar to uninfected children. The results strongly suggest that parasite load may be a key determinant of peripheral cell numbers and proportions during malaria infections.

Two measures of parasite load were used in this study: *Pf*HRP2 levels and parasite densities. Even though a strong positive correlation between *Pf*HRP2 levels and parasite densities was seen in the combined data of symptomatic and asymptomatic children, it is noteworthy that results of other studies directly addressing the correlation between parasite density and *Pf*HRP2 have been inconsistent. Some studies have demonstrated a strong relationship [32], whereas others found no correlation [33]. *Pf*HRP2 concentrations are thought to be more representative of the total parasite load in the body because *Pf*HRP2 can be released from both sequestered and circulating parasites [34]. When the ratio of sequestered to circulating parasites is relatively constant, then *Pf*HRP2 and parasite density will correlate well. However, when a greater proportion of parasites are sequestered, as has been described in some forms of severe malaria, then *Pf*HRP2 may be a better measure of parasite load [35].

Acute symptomatic malaria infection is characterized by a rapid reduction of peripheral blood lymphocyte subsets: CD4 + , CD8 + , CD3 + T cells, and CD19 + B cells [25]. These cells play a critical but complex role in parasite clearance and disease progression. Malaria parasites tend to sequester for a large portion of the asexual stage, a mechanism that has been shown to favour parasite growth by evading the filtering activity of the spleen [36]. Accordingly, to carry out their anti-malarial effector functions, it is obvious that lymphocytes will continuously recirculate from the peripheral blood and patrol other body parts and tissues where they may be required [37]. Studies in animal models and humans revealed that cytokine production is increased during acute malaria infection with consequent induction of fever and other malaria-related symptoms [23, 38, 39]. It is noteworthy that high cytokine production correlates with increased expression of adhesion molecules on endothelial cells [40] and leucocytes [41]. In turn, activated endothelial cells will interact and entrap circulating leucocytes into the microvasculature of organs and this is a crucial step in inflammation. Indeed, it has been shown that IFN-γ, which is significantly elevated in acute malaria as compared to asymptomatic infections [42], drastically caused the retrafficking of leucocytes into lymph nodes [43]. Similarly, mice lacking the α-chain of the IFN-γ receptor were characterized by the absence of leucocyte accumulation in the brain vasculature [44]. Therefore, the loss of peripheral blood lymphocytes subpopulations observed in the symptomatic participants may be due to an enhanced tissue infiltration and attachment [25]. Alternatively, abnormal cell death through apoptosis has also been indicated as another mechanism by which lymphocytes are depleted from blood during acute malaria infections [45]. This explanation is corroborated by the observation of high expression of exhaustion and senescence markers on T cells from children with symptomatic malaria compared to asymptomatic participants [46, 47]. It is becoming increasingly clear that many chronic human infectious diseases, including *P. falciparum* infections [48, 49] and HIV [50], are associated with the accumulation of atypical memory B cells that are characterized by the expression of exhausted markers, and a reduction of [51]. Although the present study did not look for atypical memory B cells, it has been demonstrated that peripheral B cells of both asymptomatic and uninfected children living in malaria endemic settings were dominated by atypical memory B cells [48]. Although atypical memory B cells are phenotypically exhausted, a recent study has shown increased expression of these cells to be associated with greater protection against malaria [52]. Thus, further work is needed to ascertain the roles of exhausted immune cells in asymptomatic *versus* symptomatic malaria infections.

The dynamic regulation of the number of monocytes and their activation status is crucial in determining infection outcome [53]. The effect of the parasite on changes of circulating monocytes remains unclear. While some studies have reported increased monocytes count during asymptomatic infections relative to noninfected control [54], others did not find any differences in monocytes counts between the two groups [53]. Consequently, the proportions and count of monocytes were the same amongst the groups in the present study. Nonetheless, a positive correlation was observed between parasite densities and both the absolute numbers and the proportions of CD14 + monocytes in the asymptomatic group. Further work is needed to characterize different subtypes (classical, intermediate and nonclassical) of monocytes, since changes in the numbers of these have been implicated in infection outcomes with *P. falciparum* infection [55].

It is intriguing to see different changes in human immune cells in the different clinical states of malaria. Whereas CD4 + , CD8 + , CD3 + , and CD19 + lymphocytes were markedly decreased in patients with acute malaria infections, the percentages and absolute count of CD15 + neutrophils were significantly expanded compared to asymptomatically infected patients. Interestingly, the proportion of CD15 + neutrophils tend to be higher in the asymptomatic group than the control uninfected group although this difference was not significant. A strong positive association was observed between the percentages and absolute cell counts of neutrophil and parasitaemia detected in the symptomatic participants. Whether increased neutrophil numbers promote parasitaemia [56] or higher parasite load drives neutrophil expansion is not certain at this point. However, the findings imply a possible link between the two. The elevation of neutrophils in proportion to disease severity also suggests that increased neutrophil numbers may contribute to malaria clinical manifestations. This is in support of a previous study showing that depletion of neutrophils prevented the development of cerebral malaria in mice [57]. Similarly in humans, neutrophil granule protein genes were increased across all severe malaria cases [35]. Given that increased neutrophils and high parasitaemia are both associated with developing clinical consequences of malaria [35, 57, 58], it is likely that the presence of a direct relationship between neutrophil cell count and parasite load in the acute *Plasmodium* infections may explain why these individuals showed symptoms. However, the absence of association between neutrophils and parasitaemia in asymptomatic infections suggests that neutrophil expansion and the production of neutrophil related cytokines are tightly regulated in the asymptomatic individuals favouring parasite clearance while at the same time avoiding clinical consequences from extensive neutrophilia. It will be interesting to determine whether, beyond the differences in absolute counts, if neutrophils from the different study groups exhibit different phenotypes.

In this study, no changes was observed in the frequencies and absolute count of γδ T cells in any of the study groups, which agreed with a previous study conducted in children [28]. In contrast, other studies have shown the frequency of γδ T cells to markedly expand during acute *Plasmodium* infections in naïve individuals [24]. Interestingly, Jagannathan et al*.* have shown that γδ T lymphocyte cells lose their ability to proliferate following repeated exposure [14] and this could explain why the present study found no change in the frequency of this cell type. There is a high possibility that the participants in this study may have had repeated previous parasite exposure, suggesting that the γδ T cells may have lost the ability to proliferate upon parasite stimulation. Of course, the possibility of tissue sequestration playing a critical role in determining the outcome of peripheral frequency of γδ T cells in malaria infections cannot be ruled out [59].

## Conclusion

This study presents a characterization of the peripheral blood leucocyte subpopulations in children with acute symptomatic or asymptomatic *P. falciparum* malaria, as well as uninfected children in Obom, Accra, Ghana. Given the observational nature of this study and the fact that there remain other unmeasured factors which differ between the groups and might contribute to the differences in leucocyte populations, the authors are cautious in drawing conclusions. However, the present data demonstrates quite clearly that while symptomatic malaria results in peripheral lymphopenia and neutrophilia in a parasite dependent manner, the peripheral blood composition in asymptomatic individuals resemble very closely those in healthy controls. This would appear to support the notion that clinical immunity to malaria involves both the control of parasite load and tolerance to low-level parasitaemia [39, 60].

## Supplementary information

**Additional file 1: Figure S1.** Flow cytometric strategy used to identify leucocyte subsets.

**Additional file 2: Figure S2.** Absolute counts of leucocytes in the peripheral blood of Ghanaian children who are uninfected (n = 15), or with asymptomatic (n = 52) or symptomatic (n = 22) *P. falciparum* infections.

**Additional file 3: Figure S3.** Relationships between parasite biomass (*Pf*HRP2 plasma levels) and absolute numbers of different cell subsets in the peripheral blood of *Pf*-infected children.

**Additional file 4: Figure S4.** Relationships between parasite densities and absolute numbers of different cell subsets in the peripheral blood of *Pf*-infected children.

## Data Availability

The datasets during and/or analysed during the current study available from the corresponding author on reasonable request.

## References

[1] WHO (2019). World malaria report 2019.

[2] Ashley EA, Dhorda M, Fairhurst RM, Amaratunga C, Lim P, Suon S (2014). Spread of artemisinin resistance in *Plasmodium falciparum* malaria. N Engl J Med.

[3] Leang R, Taylor WR, Bouth DM, Song L, Tarning J, Char MC (2015). Evidence of *Plasmodium falciparum* malaria multidrug resistance to artemisinin and piperaquine in western Cambodia: dihydroartemisinin-piperaquine open-label multicenter clinical assessment. Antimicrob Agents Chemother.

[4] Ranson H, Lissenden N (2016). Insecticide resistance in African *Anopheles* mosquitoes: a worsening situation that needs urgent action to maintain malaria control. Trends Parasitol.

[5] Gowda DC, Wu X (2018). Parasite recognition and signaling mechanisms in innate immune responses to malaria. Front Immunol.

[6] Langhorne J, Ndungu FM, Sponaas A-M, Marsh K (2008). Immunity to malaria: more questions than answers. Nat Immunol.

[7] Doolan DL, Dobaño C, Baird JK (2009). Acquired immunity to malaria. Clin Microbiol Rev.

[8] Wilairatana P, Tangpukdee N, Krudsood S (2013). Definition of hyperparasitemia in severe falciparum malaria should be updated. Asian Pac J Trop Biomed.

[9] Buffet PA, Safeukui I, Deplaine G, Brousse V, Prendki V, Thellier M (2011). The pathogenesis of *Plasmodium falciparum* malaria in humans: insights from splenic physiology. Blood.

[10] Chen Q, Amaladoss A, Ye W, Liu M, Dummler S, Kong F (2014). Human natural killer cells control *Plasmodium falciparum* infection by eliminating infected red blood cells. Proc Natl Acad Sci USA.

[11] Langhorne J, Gillard S, Simon B, Slade S, Eichmann K (1989). Frequencies of CD4+ T cells reactive with *Plasmodium chabaudi chabaudi*: distinct response kinetics for cells with Th1 and Th2 characteristics during infection. Int Immunol.

[12] Hojo-Souza NS, Pereira DB, Passos LSA, Gazzinelli-Guimarães PH, Cardoso MS, Tada MS (2015). Phenotypic profiling of CD8+ T cells during *Plasmodium vivax* blood-stage infection. BMC Infect Dis.

[13] Riggle BA, Manglani M, Maric D, Johnson KR, Lee MH, Neto OLA (2020). CD8+ T cells target cerebrovasculature in children with cerebral malaria. J Clin Invest.

[14] Jagannathan P, Lutwama F, Boyle MJ, Nankya F, Farrington LA, McIntyre TI (2017). Vδ2+ T cell response to malaria correlates with protection from infection but is attenuated with repeated exposure. Sci Rep.

[15] Blackman MJ, Heidrich H-G, Donachie S, McBride J, Holder A (1990). A single fragment of a malaria merozoite surface protein remains on the parasite during red cell invasion and is the target of invasion-inhibiting antibodies. J Exp Med.

[16] Hill DL, Eriksson EM, Suen CSLW, Chiu CY, Ryg-Cornejo V, Robinson LJ (2013). Opsonising antibodies to *P. falciparum* merozoites associated with immunity to clinical malaria. PLoS ONE.

[17] Arora G, Hart GT, Manzella-Lapeira J, Doritchamou JY, Narum DL, Thomas LM (2018). NK cells inhibit *Plasmodium falciparum* growth in red blood cells via antibody-dependent cellular cytotoxicity. eLife..

[18] Osier FH, Fegan G, Polley SD, Murungi L, Verra F, Tetteh KK (2008). Breadth and magnitude of antibody responses to multiple *Plasmodium falciparum* merozoite antigens are associated with protection from clinical malaria. Infect Immun.

[19] Kolaczkowska E, Kubes P (2013). Neutrophil recruitment and function in health and inflammation. Nat Rev Immunol.

[20] Knackstedt SL, Georgiadou A, Apel F, Abu-Abed U, Moxon CA, Cunnington AJ (2019). Neutrophil extracellular traps drive inflammatory pathogenesis in malaria. Sci Immunol.

[21] Amulic B, Moxon CA, Cunnington AJ (2020). A more granular view of neutrophils in malaria. Trends Parasitol.

[22] Fontana MF, de Melo GL, Anidi C, Hamburger R, Kim CY, Lee SY (2016). Macrophage colony stimulating factor derived from CD4+ T cells contributes to control of a blood-borne infection. PLoS Pathog.

[23] Walther M, Woodruff J, Edele F, Jeffries D, Tongren JE, King E (2006). Innate immune responses to human malaria: heterogeneous cytokine responses to blood-stage *Plasmodium falciparum* correlate with parasitological and clinical outcomes. J Immunol.

[24] Ho M, Tongtawe P, Kriangkum J, Wimonwattrawatee T, Pattanapanyasat K, Bryant L (1994). Polyclonal expansion of peripheral gamma delta T cells in human *Plasmodium falciparum* malaria. Infect Immun.

[25] Kassa D, Petros B, Mesele T, Hailu E, Wolday D (2006). Characterization of peripheral blood lymphocyte subsets in patients with acute *Plasmodium falciparum* and *P. vivax* malaria infections at Wonji Sugar Estate, Ethiopia. Clin Vaccine Immunol..

[26] Roussilhon C, Agrapart M, Ballet J-J, Bensussan A (1990). T lymphocytes bearing the γδ T cell receptor in patients with acute *Plasmodium falciparum* malaria. J Infect Dis.

[27] Worku S, Björkman A, Troye‐Blomberg M, Jemaneh L, Färnert A, Christensson B. Lymphocyte activation and subset redistribution in the peripheral blood in acute malaria illness: distinct γδ+ T cell patterns in *Plasmodium falciparum* and *P. vivax* infections. Clin Exp Immunol. 1997;108:34–41. 10.1046/j.1365-2249.1997.d01-981.x PMC1904634 9097908

[28] Hviid L, Kurtzhals J, Dodoo D, Rodrigues O, Rønn A, Commey J (1996). The gamma/delta T-cell response to *Plasmodium falciparum* malaria in a population in which malaria is endemic. Infect Immun.

[29] Lisse IM, Aaby P, Whittle H, Knudsen K (1994). A community study of T lymphocyte subsets and malaria parasitaemia. Trans R Soc Trop Med Hyg.

[30] Amoah LE, Acquah FK, Ayanful-Torgby R, Oppong A, Abankwa J, Obboh EK (2018). Dynamics of anti-MSP3 and Pfs230 antibody responses and multiplicity of infection in asymptomatic children from southern Ghana. Parasit Vectors.

[31] Adjah J, Fiadzoe B, Ayanful-Torgby R, Amoah LE (2018). Seasonal variations in Plasmodium falciparum genetic diversity and multiplicity of infection in asymptomatic children living in southern Ghana. BMC infect Dis.

[32] Desakorn V, Dondorp AM, Silamut K, Pongtavornpinyo W, Sahassananda D, Chotivanich K (2005). Stage-dependent production and release of histidine-rich protein 2 by *Plasmodium falciparum*. Trans R Soc Trop Med Hyg.

[33] Pava Z, Echeverry DF, Díaz G, Murillo C (2010). Large variation in detection of histidine-rich protein 2 in *Plasmodium falciparum* isolates from Colombia. Ame J Trop Med Hyg.

[34] Kifude CM, Rajasekariah HG, Sullivan DJ, Stewart VA, Angov E, Martin SK (2008). Enzyme-linked immunosorbent assay for detection of *Plasmodium falciparum* histidine-rich protein 2 in blood, plasma, and serum. Clin Vaccine Immunol.

[35] Lee HJ, Georgiadou A, Walther M, Nwakanma D, Stewart LB, Levin M (2018). Integrated pathogen load and dual transcriptome analysis of systemic host-pathogen interactions in severe malaria. Sc Transl Med.

[36] Deroost K, Pham T-T, Opdenakker G, Van den Steen PE (2015). The immunological balance between host and parasite in malaria. FEMS Microbiol Rev.

[37] Rosenberg YJ, Cafaro A, Brennan T, Greenhouse JG, Villinger F, Ansari AA (1997). Virus-induced cytokines regulate circulating lymphocyte levels during primary SIV infections. Int Immunol.

[38] Kern P, Hemmer CJ, Van Damme J, Gruss H-J, Dietrich M (1989). Elevated tumor necrosis factor alpha and interleukin-6 serum levels as markers for complicated *Plasmodium falciparum* malaria. Am J Med.

[39] Ademolue TW, Aniweh Y, Kusi KA, Awandare GA (2017). Patterns of inflammatory responses and parasite tolerance vary with malaria transmission intensity. Malar J.

[40] Chang Y-J, Holtzman MJ, Chen C-C (2002). Interferon-γ-induced epithelial ICAM-1 expression and monocyte adhesion involvement of protein kinase c-dependent c-Src tyrosine kinase activation pathway. J Biol Chem.

[41] Del Pozo MA, Sánchez-Mateos P, Nieto M, Sánchez-Madrid F (1995). Chemokines regulate cellular polarization and adhesion receptor redistribution during lymphocyte interaction with endothelium and extracellular matrix. Involvement of cAMP signaling pathway. J Cell Biol.

[42] Iriemenam NC, Okafor C, Balogun HA, Ayede I, Omosun Y, Persson J-O (2009). Cytokine profiles and antibody responses to *Plasmodium falciparum* malaria infection in individuals living in Ibadan, southwest Nigeria. Afr Health Sci.

[43] Westermann J, Persin S, Matyas J, van der Meide P, Pabst R (1993). IFN-gamma influences the migration of thoracic duct B and T lymphocyte subsets in vivo. Random increase in disappearance from the blood and differential decrease in reappearance in the lymph. J Immunol.

[44] Amani V, Vigário AM, Belnoue E, Marussig M, Fonseca L, Mazier D (2000). Involvement of IFN-γ receptor-mediated signaling in pathology and anti-malarial immunity induced by *Plasmodium berghei* infection. Eur J Immunol.

[45] Helmby H, Jönsson G, Troye-Blomberg M (2000). Cellular changes and apoptosis in the spleens and peripheral blood of mice infected with blood-stage *Plasmodium chabaudi chabaudi* AS. Infect Immun.

[46] Frimpong A, Kusi KA, Adu-Gyasi D, Amponsah J, Ofori MF, Ndifon W (2019). Phenotypic evidence of T cell exhaustion and senescence during symptomatic *Plasmodium falciparum* Malaria. Front Immunol.

[47] Illingworth J, Butler NS, Roetynck S, Mwacharo J, Pierce SK, Bejon P (2013). Chronic exposure to *Plasmodium falciparum* is associated with phenotypic evidence of B and T cell exhaustion. J Immunol.

[48] Weiss GE, Crompton PD, Li S, Walsh LA, Moir S, Traore B (2009). Atypical memory B cells are greatly expanded in individuals living in a malaria-endemic area. J Immunol.

[49] Ubillos I, Campo JJ, Requena P, Ome-Kaius M, Hanieh S, Rose H (2017). Chronic exposure to malaria is associated with inhibitory and activation markers on atypical memory B cells and marginal zone-like B cells. Front Immunol.

[50] Moir S, Ho J, Malaspina A, Wang W, DiPoto AC, O'Shea MA (2008). Evidence for HIV-associated B cell exhaustion in a dysfunctional memory B cell compartment in HIV-infected viremic individuals. J Exp Med.

[51] Portugal S, Tipton CM, Sohn H, Kone Y, Wang J, Li S (2015). Malaria-associated atypical memory B cells exhibit markedly reduced B cell receptor signaling and effector function. Elife.

[52] Sullivan RT, Ssewanyana I, Wamala S, Nankya F, Jagannathan P, Tappero JW (2016). B cell sub-types following acute malaria and associations with clinical immunity. Malar J.

[53] Dobbs KR, Embury P, Vulule J, Odada PS, Rosa BA, Mitreva M (2017). Monocyte dysregulation and systemic inflammation during pediatric falciparum malaria. JCI Insight.

[54] Gansane A, Ouedraogo IN, Henry NB, Soulama I, Ouedraogo E, Yaro J-B (2013). Variation in haematological parameters in children less than five years of age with asymptomatic *Plasmodium* infection: implication for malaria field studies. Mem Ins Oswaldo Cruz.

[55] Royo J, Rahabi M, Kamaliddin C, Ezinmegnon S, Olagnier D, Authier H (2019). Changes in monocyte subsets are associated with clinical outcomes in severe malarial anaemia and cerebral malaria. Sci Rep.

[56] Otterdal K, Berg A, Michelsen AE, Patel S, Tellevik MG, Haanshuus CG (2018). Soluble markers of neutrophil, T-cell and monocyte activation are associated with disease severity and parasitemia in falciparum malaria. BMC Infect Dis.

[57] Chen L, Zhang ZH, Sendo F (2000). Neutrophils play a critical role in the pathogenesis of experimental cerebral malaria. Clin Exp Immunol.

[58] Sercundes MK, Ortolan LS, Debone D, Soeiro-Pereira PV, Gomes E, Aitken EH (2016). Targeting neutrophils to prevent malaria-associated acute lung injury/acute respiratory distress syndrome in mice. PLoS Path.

[59] Langhorne J, Simon-Haarhaus B (1991). Differential T cell responses to *Plasmodium chabaudi chabaudi* in peripheral blood and spleens of C57BL/6 mice during infection. J Immunol.

[60] Ademolue TW, Awandare GA (2018). Evaluating antidisease immunity to malaria and implications for vaccine design. Immunoly.

