# Supplementary figures and images for: Comparison of leucocyte profiles between healthy children and those with asymptomatic and symptomatic Plasmodium falciparum infections
Source: Malar J. 2020 Oct 9;19:364. doi: 10.1186/s12936-020-03435-x (PMC7547495; doi:10.1186/s12936-020-03435-x)

# Viable

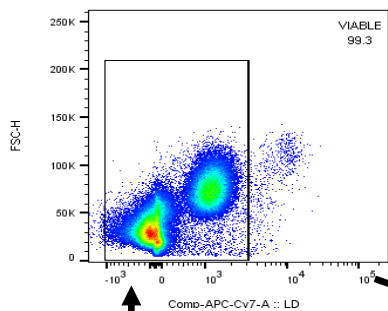

## singlet

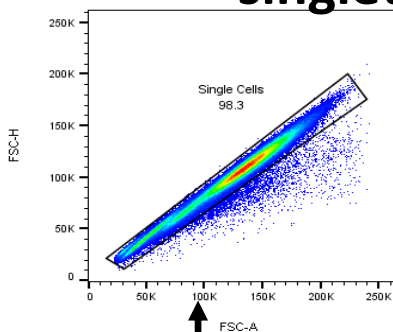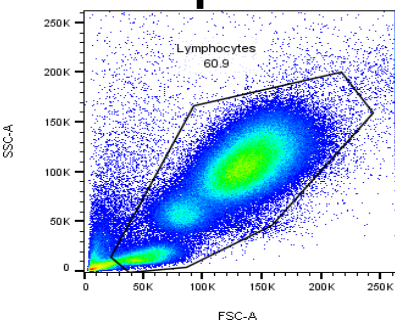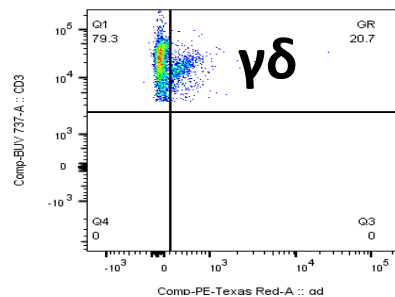

## CD8

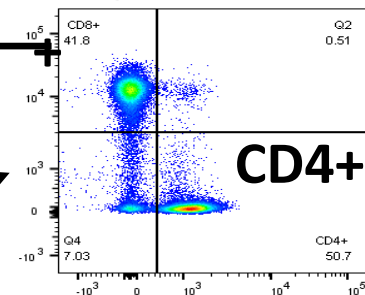

## CD3+

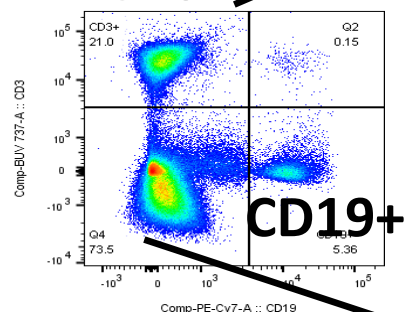

## CD11c+

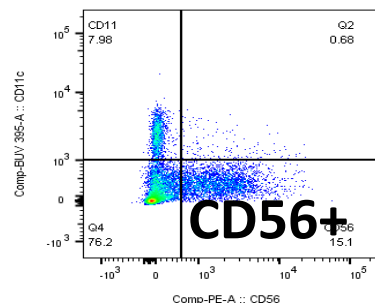

## CD14+

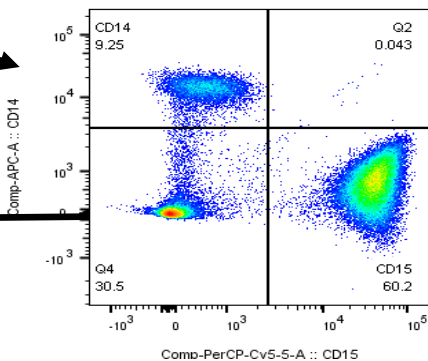

## CD15+

Supplement: Supplementary file 1 — Additional file 1: Figure S1. Flow cytometric strategy used to identify leucocyte subsets. [file 12936_2020_3435_MOESM1_ESM.pdf]

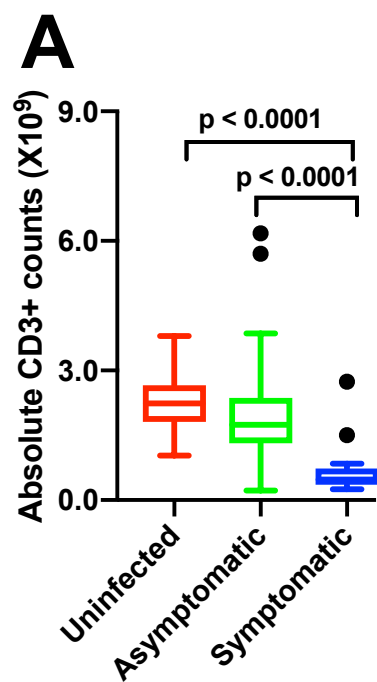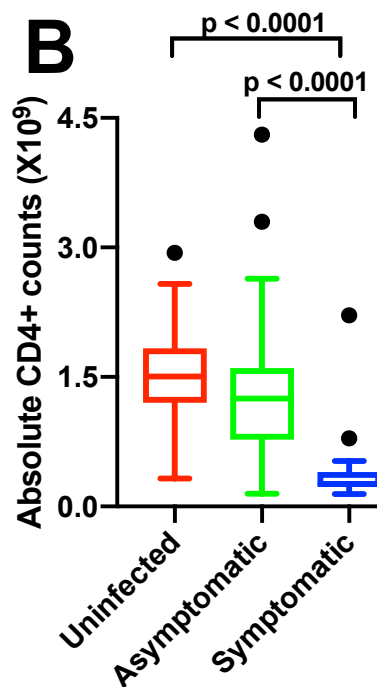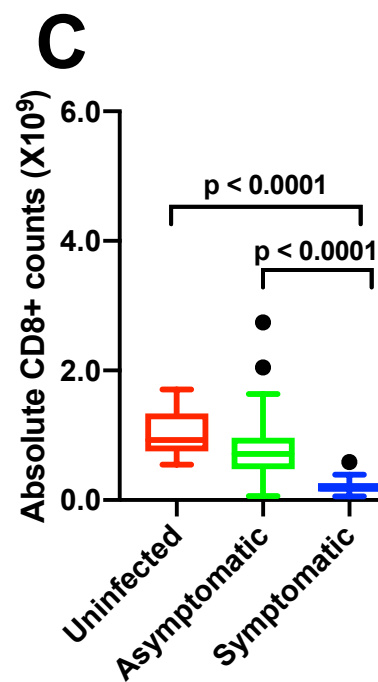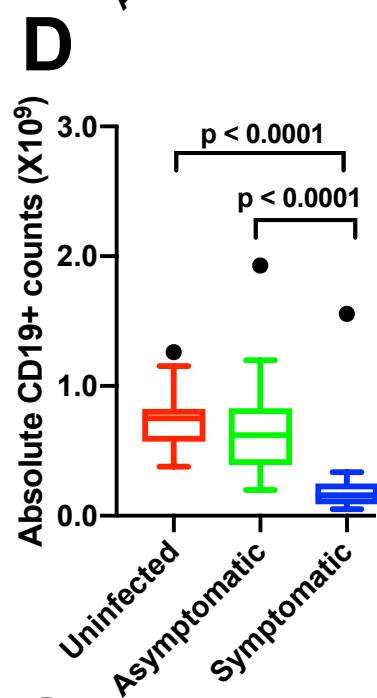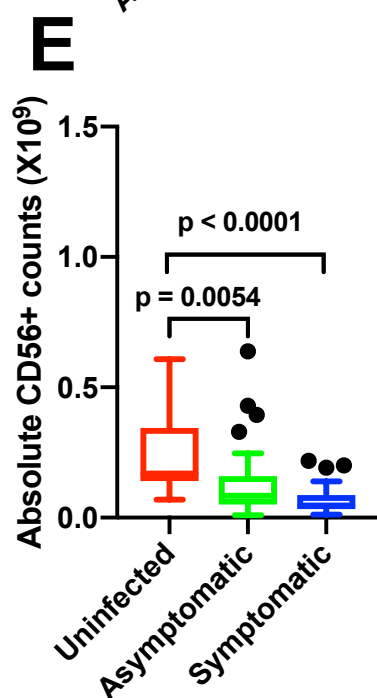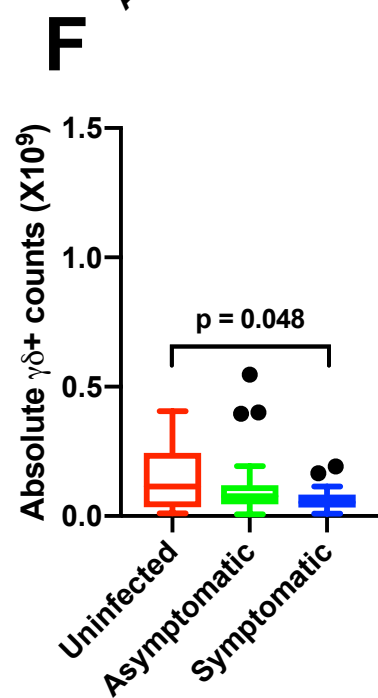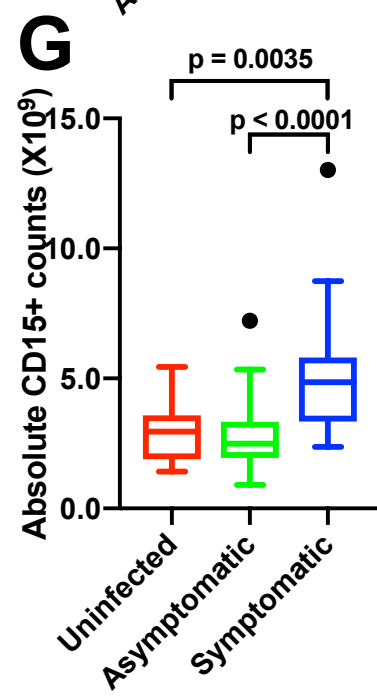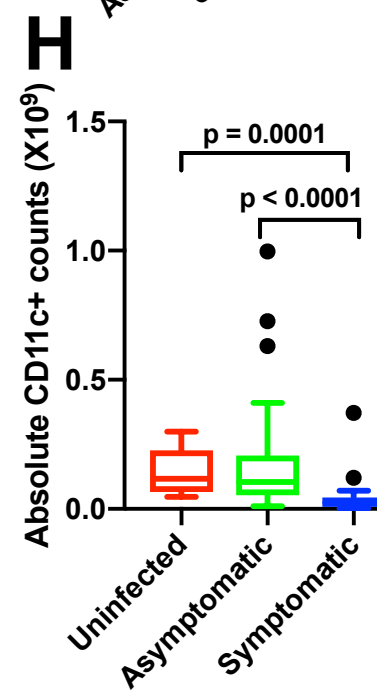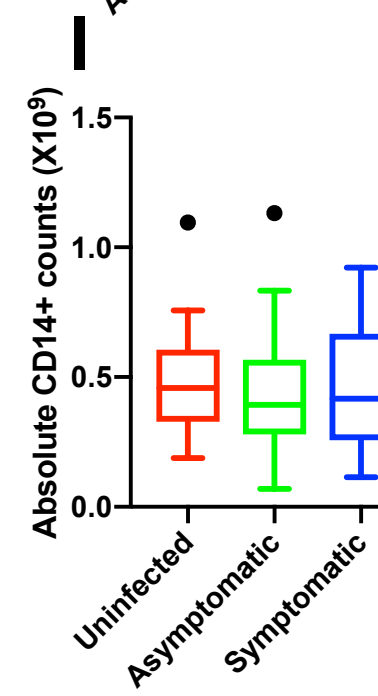

Supplement: Supplementary file 2 — Additional file 2: Figure S2. Absolute counts of leucocytes in the peripheral blood of Ghanaian children who are uninfected (n = 15), or with asymptomatic (n = 52) or symptomatic (n = 22) P. falciparum infections. [file 12936_2020_3435_MOESM2_ESM.pdf]

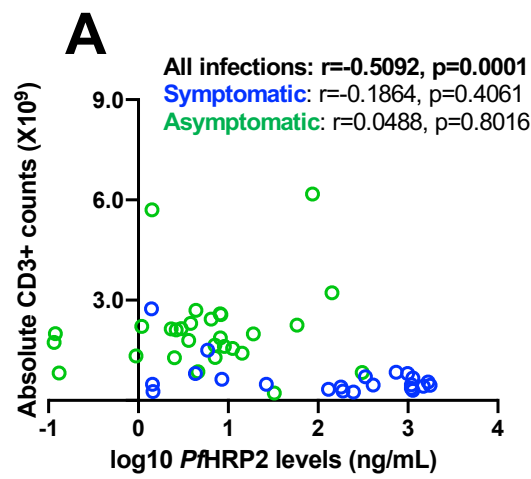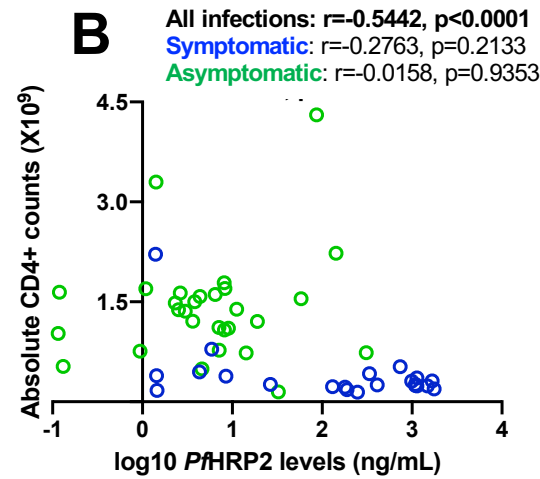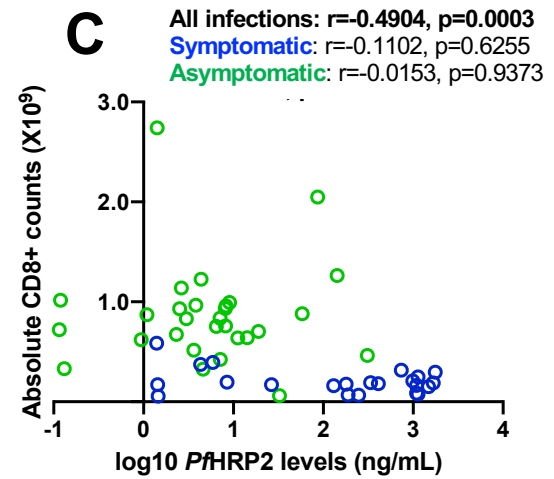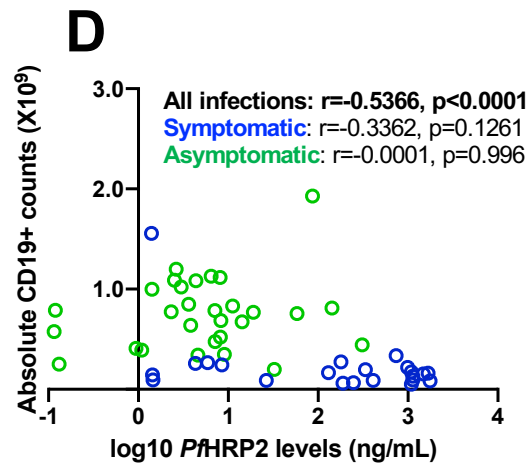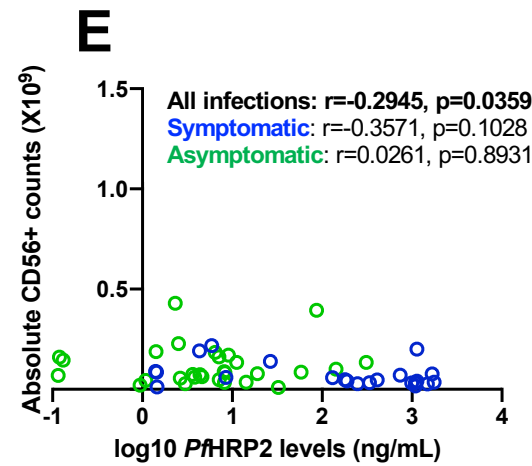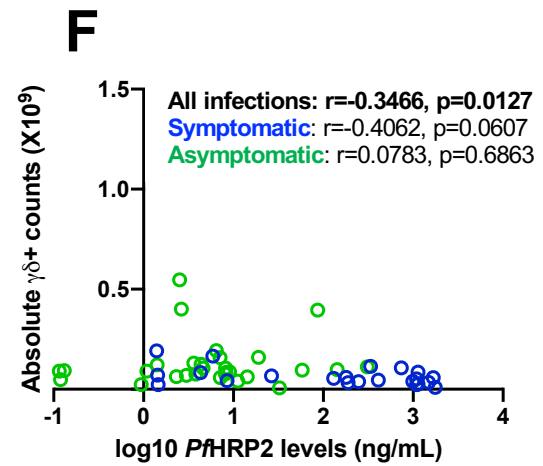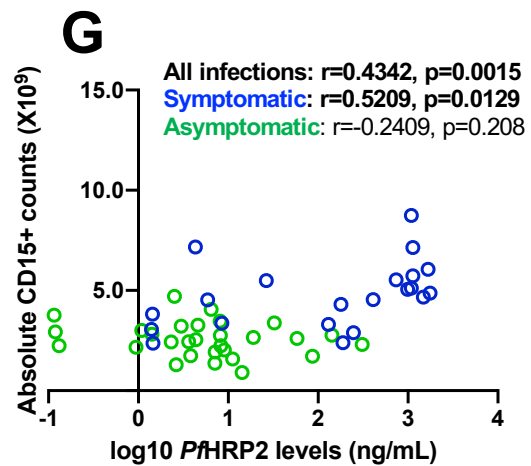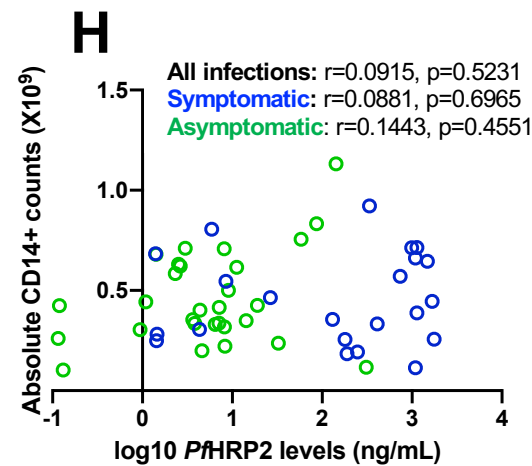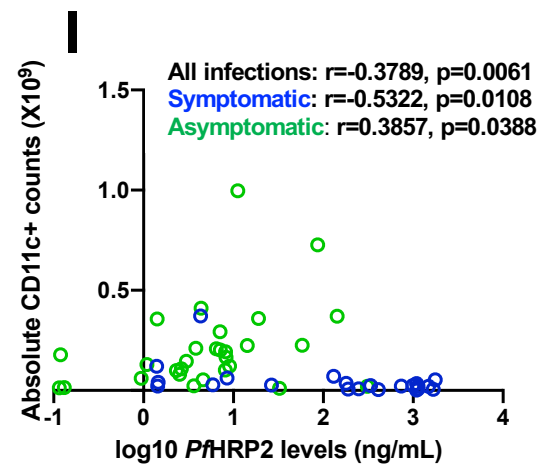

Supplement: Supplementary file 3 — Additional file 3: Figure S3. Relationships between parasite biomass (PfHRP2 plasma levels) and absolute numbers of different cell subsets in the peripheral blood of Pf-infected children. [file 12936_2020_3435_MOESM3_ESM.pdf]

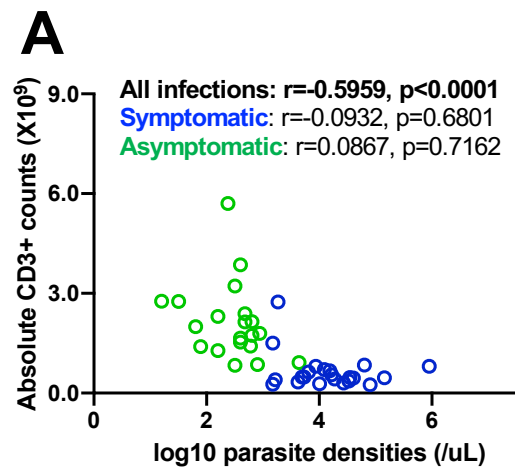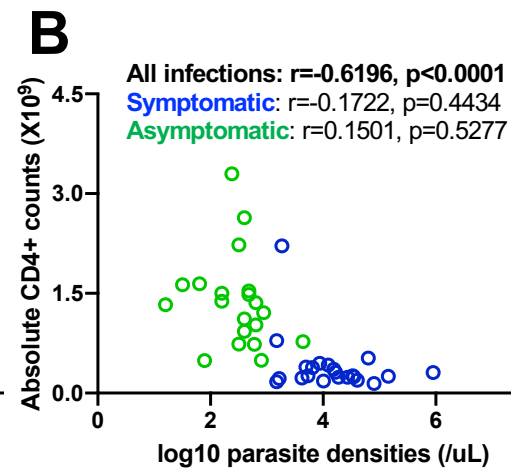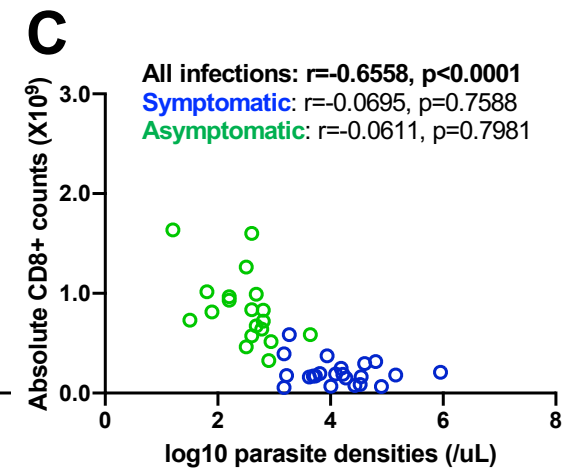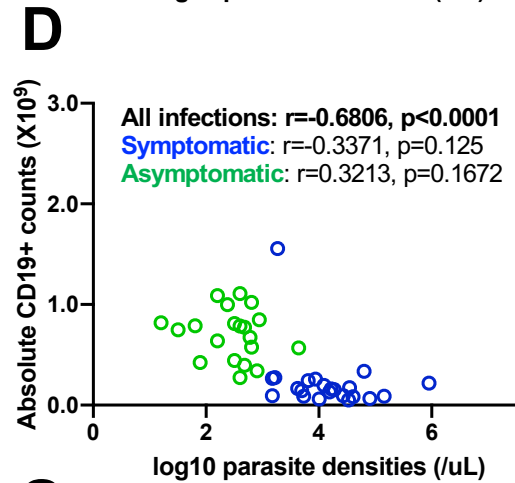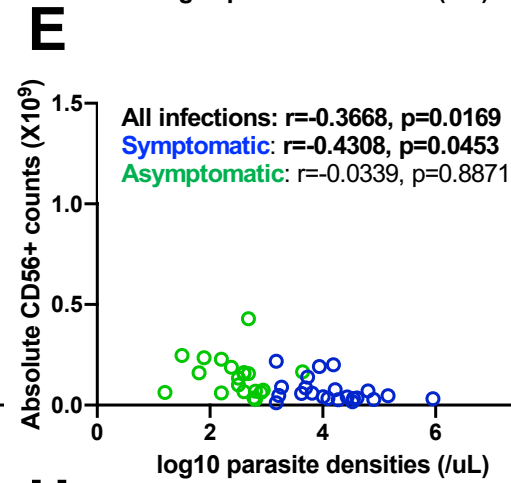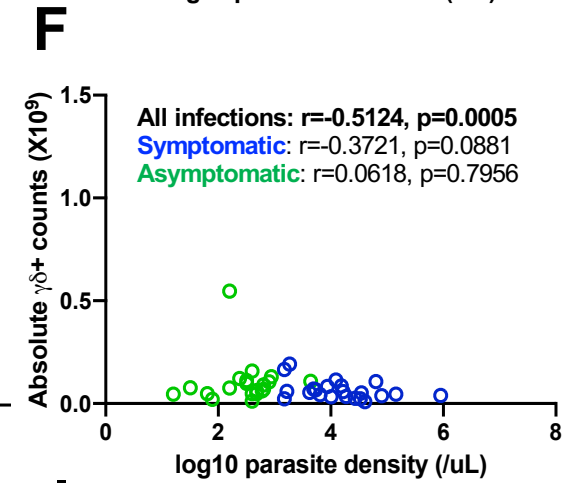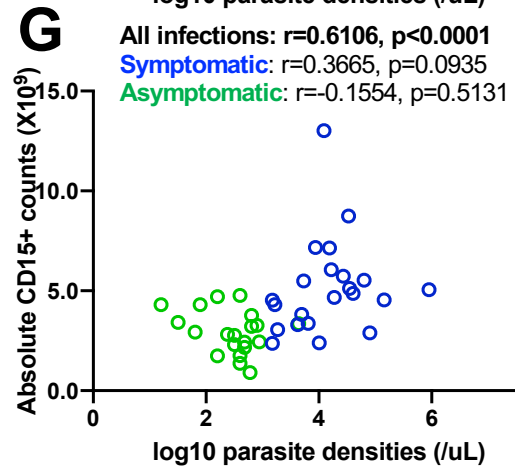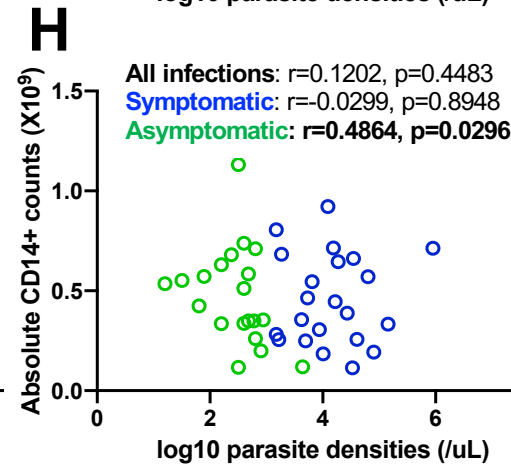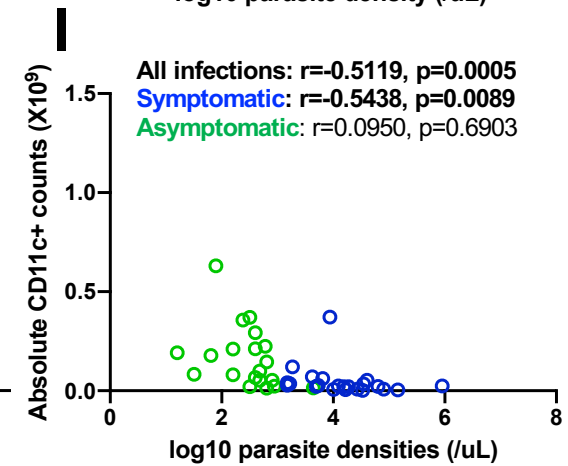

Supplement: Supplementary file 4 — Additional file 4: Figure S4. Relationships between parasite densities and absolute numbers of different cell subsets in the peripheral blood of Pf-infected children. [file 12936_2020_3435_MOESM4_ESM.pdf]
